# Effects of reducing free sugars on 24-hour glucose profiles and glycemic variability in subjects without diabetes

**DOI:** 10.3389/fnut.2023.1213661

**Published:** 2023-10-02

**Authors:** Christina Laeticia Pappe, Beeke Peters, Henrik Dommisch, Johan Peter Woelber, Olga Pivovarova-Ramich

**Affiliations:** ^1^Department of Periodontology, Oral Medicine and Oral Surgery, Charité – Universitätsmedizin Berlin, Corporate Member of Freie Universität Berlin, Humboldt-Universität zu Berlin, and Berlin Institute of Health, Berlin, Germany; ^2^Research Group Molecular Nutritional Medicine and Department of Human Nutrition, German Institute of Human Nutrition Potsdam-Rehbruecke, Nuthetal, Germany; ^3^German Center for Diabetes Research (DZD), Oberschleißheim, Germany; ^4^Department of Periodontology, Health Science Center, University of Washington, Seattle, WA, United States; ^5^Policlinic of Operative Dentistry, Periodontology, and Pediatric Dentistry, Medical Faculty Carl Gustav Carus, Technische Universität Dresden, Dresden, Germany; ^6^Department of Endocrinology, Diabetes and Nutrition, Campus Benjamin Franklin, Charité-Universitätsmedizin Berlin, Freie Universität Berlin, Humboldt-Universität zu Berlin, Berlin Institute of Health, Berlin, Germany

**Keywords:** free sugar reduction, glucose metabolism, continuous glucose monitoring, glycemic variability, obesity

## Abstract

**Background:**

The Western diet, especially beverages and high processed food products, is high in sugars which are associated with the development of obesity and diabetes. The reduction of refined carbohydrates including free and added sugars improves glycemic control in individuals with diabetes, but the data regarding effects in subjects without diabetes are limited.

**Objective:**

This study aimed to evaluate the effects of reducing free sugar intake on 24-h glucose profiles and glycemic variability using continuous glucose monitoring (CGM).

**Methods:**

In the randomized controlled study, 21 normal weight and overweight/obese subjects (BMI 18–40 kg/m^2^) without diabetes were assigned to a 4-week reduced-sugar (RS) diet or control diet after a 2-week baseline phase. During the baseline phase, all participants were advised not to change their habitual diet. During the intervention phase, RS participants were asked to avoid added sugar and white flour products, whereas participants of the control group were requested to proceed their habitual diet. Anthropometric parameters and HbA1c were assessed before and at the end of the intervention phase. Interstitial glucose was measured using continuous glucose monitoring (CGM), and the food intake was documented by dietary records for 14 consecutive days during the baseline phase and for the first 14 consecutive days during the intervention phase. Mean 24-h glucose as well as intra- and inter-day indices of glucose variability, i.e., standard deviation (SD) around the sensor glucose level, coefficient of variation in percent (CV), mean amplitude of glucose excursions (MAGE), continuous overlapping net glycemic action (CONGA), and mean absolute glucose (MAG), were calculated for the baseline and intervention phases.

**Results:**

During the intervention, the RS group decreased the daily intake of sugar (i.e., −22.4 ± 20.2 g, −3.28 ± 3.61 EN %), total carbohydrates (−6.22 ± 6.92 EN %), and total energy intake (−216 ± 108 kcal) and increased the protein intake (+2.51 ± 1.56 EN %) compared to the baseline values, whereby this intervention-induced dietary changes differed from the control group. The RS group slightly reduced body weight (−1.58 ± 1.33 kg), BMI, total fat, and visceral fat content and increased muscle mass compared to the baseline phase, but these intervention-induced changes showed no differences in comparison with the control group. The RS diet affected neither the 24-h mean glucose levels nor intra- and inter-day indices of glucose variability, HbA1c, or diurnal glucose pattern in the within- and between-group comparisons.

**Conclusion:**

The dietary reduction of free sugars decreases body weight and body fat which may be associated with reduced total energy intake but does not affect the daily mean glucose and glycemic variability in individuals without diabetes.

**Clinical trial registration:**

German Clinical Trials Register (DRKS); identifier: DRKS00026699.

## Introduction

With the worldwide diabetes epidemic, diabetes risk factors and preventive approaches need to be more focused. Industrial revolution was associated with a global increase in sugar availability and consumption (1–3). Western style diet, which is rich in free sugars (4) in addition to high amount of saturated fat and low content of micronutrients, clearly contributes to the obesity and diabetes epidemic in Western society (5). In particular, excessive consumption of sugar-sweetened beverages is associated with an increasing risk of type 2 diabetes (T2D) (3, 6).

For this reason, the World Health Organization (WHO) recommends a reduction of the free sugar intake to <10%, ideally under 5% of daily energy intake (EN%) (3, 7). The WHO defines free sugars as a sum of (i) mono- and disaccharides in food and beverages which are added by the manufacturer, consumer, or cook (designated as “added sugars”) and (ii) sugars in fruit and vegetable juices, juice concentrates, and those naturally present in honey or syrup (7, 8). Notably, most studies cited below analyzed the health impact of added sugars and did not consider foods naturally containing high sugar amounts although free sugar consumption through these might even be higher than the added sugar amounts (8).

Increased sugar consumption has led to controversial debates about its health effects (3, 9). In a systemic review and meta-analysis of 30 trials and 38 cohort studies in children and adults eating *ad libitum*, the intake of free sugars and/or sugar-sweetened beverages was highlighted as a determinant of body weight, whereas the reduction of dietary sugars was associated with weight loss probably due to the change in energy intake (10). Regarding body weight and fat gain, high intake of free sugar is discussed to contribute to the development of T2D as well as cardiovascular diseases (CVD) independently from calorie intake and weight gain (9, 11). Interestingly, a study investigated sugar availability in different countries and revealed its association with diabetes prevalence (11). Nevertheless, the evidence for adverse health effects, e.g., obesity, T2D, and CVD due to high added sugar intake, is limited, and the recommendations for sugar intake are therefore heterogenic (12), leading to an increased demand for more evidence-based guidelines (13).

In general, high sugar intake leads to an increased rise of postprandial glucose (PPG). In a multicenter study in 3,284 men and women with non-treated T2D, postprandial increase of glucose levels was described as a frequent phenomenon in patients with T2D (14). Similar observations were made in subjects with impaired glucose tolerance (15). High PPG is a predominant contributor to the overall diurnal hyperglycemia in T2D (16) and correlates with T2D morbidity and cardiovascular mortality (17, 18). Glycemic variability (GV; the amplitude, frequency, and duration of glycemic fluctuations around mean blood glucose) is an independent risk factor for diabetes-related complications, including micro- and macro-vascular complications, in people with diabetes and therefore represents an emerging target for blood glucose control (19). Even in people without diabetes, increased GV is a predictor of cardiovascular complications (20). Several studies showed that glycemic control in T2D can be improved by the dietary reduction of total carbohydrate intake, especially of high glycemic index carbohydrates and added sugars (21–23). This effect appears to be mediated, at least partly, by the reduction of PPG. Through the development and increasing availability of continuous glucose monitoring (CGM) systems, a range of GV indices can be analyzed to assess dietary effects on different aspects of intra- and inter-day glucose variability in T2D (24). However, whether the reduction of free sugar intake can also improve glycemic control in subjects without diabetes is unknown. Therefore, the aim of this study was to evaluate the effects of reducing free sugar intake on the 24-h mean glucose levels and GV in subjects without diabetes using CGM.

## Materials and methods

### Study design

A total of 22 patients with a generalized mild-to-moderate periodontitis completed the dietary intervention study in addition to standard periodontal therapy. A randomized parallel-arm controlled trial was conducted in two groups of subjects: a group that followed a reduced-sugar (RS) diet and a control group. The study was designed as a dental study so that participants were randomized for the primary dental outcome parameter bleeding on probing index using STATA (version 17.0, College Station, TX, USA). Here, we focus on the secondary study outcomes related to the glycemic control.

During the baseline phase, all participants were advised not to change their habitual diet. They had to document their food intake by dietary records, and interstitial glucose levels were measured by continuous glucose monitoring (CGM) for 14 consecutive days. After the baseline, the intervention group followed a 4-week RS diet guided by an extensive nutrition counseling through a nutritionist prior to intervention start, whereas the control group did not receive any nutritional recommendations. Interstitial glucose was measured by CGM, and dietary records were collected again during the first 14 days of the intervention phase. Before and at the end of the intervention phase, anthropometric parameters and HbA1c were assessed. Participants were recruited by the Department for Periodontology, Oral Medicine, and Oral Surgery at the Charité-Universitätsmedizin Berlin, and the study was conducted between October 2021 and June 2022. The study was approved by the Ethics Committee of the Charité-Universitätsmedizin Berlin and registered in German Clinical Trials Register (DRKS; identifier: DRKS00026699). A written informed consent was obtained from all study participants prior to the study.

### Study participants and eligibility criteria

Study participants were male and female individuals, aged between 18 and 75 years. The inclusion criteria were a BMI between 18 and 40 kg/m^2^, consumption of a Western diet prior to study start, and a generalized mild-to-moderate periodontitis. The exclusion criteria were diabetes, pregnancy and breastfeeding, severe internal diseases, e.g., severe liver and kidney disease, severe heart failure, and cancer, eating disorders and severe psychiatric illness, smoking (defined as >5 cigarettes daily), abnormal dental status, e.g., periodontitis state IV grade C, orthodontic appliances, other ongoing dental treatments or xerostomia, medication with anticoagulants, antibiotics (in the last 3 months prior to study start), and cortisol (except for asthma spray) as well as the participation in other studies.

### Nutritional counseling and analysis of dietary records

At the beginning of the study, participants randomized to the RS group received nutritional counseling *via* telephone from a professional nutritionist. The duration of the counseling was 45 min and included information about the cardiometabolic risks of the Western diet and the associated consequences for oral health, e.g., caries and gingivitis. Furthermore, psychological and physical effects of glucose and fructose, e.g., dopamine release, habituation effects, and effects on blood sugar and cholesterol levels, were explained. In accordance with the general recommendation of the WHO, the study subjects were instructed to limit their consumption of free sugar as much as possible. Thereby, participants were asked to avoid added sugar and white flour products. In this context, alternatives, e.g., sugar substitutes such as xylitol and erythritol, were highlighted. Furthermore, fruit consumption was recommended as an alternative to sweets, and the positive effects of dietary fibers contained in fruits were emphasized. During the consultation, the approach of motivational interviewing (25) was used. Thus, participants were asked for ideas regarding individual approaches and solutions for reduction of sugar intake. In this context, an individualized action planning took place. Subjects were offered to contact the nutritionist at any time during the study. Participants of the control group were advised not to change their habitual diet for the duration of the study. Furthermore, the control group participants were offered a free dietary counseling after the study.

For the assessment of the study compliance, dietary records were collected for 14 days simultaneously with CGM. Participants were asked to document all consumed foods and drinks and the eating times during the baseline and intervention phases. They were instructed to weigh their food whenever possible, write down brand names, and use standard household measures (e.g., cups, glasses, tablespoon, and teaspoon) when they go out for dinner. Dietary records were analyzed for daily energy and macronutrient intake using the FDDB database (Fddb Internetportale GmbH, https://fddb.info/) as described (26). Using this database, the total sugar amount in each consumed food product was assessed, which corresponds to the label “of which sugar” (German label: “davon Zucker”) underneath the labeling for carbohydrates and including sugars in fruit, vegetables, or milk. Average calorie and macronutrient intake (as a percentage of daily energy intake) over 14 days was calculated for each study phase. Notably, an analysis of individual dietary records revealed one participant of the RS group who did not reduce the sugar intake. This participant was excluded so that a total of 21 subjects were included in the final statistical analyses.

### Anthropometric measurements

Body weight and body composition were analyzed by the scale and body analyzer BF 508 (OMRON, Mannheim, Germany). Waist and hip circumferences were assessed with a measurement tape.

### Continuous glucose monitoring and blood biochemistry

For the assessment of 24-h interstitial glucose, each participant was fitted with a sensor of the CGM system FreeStyle Libre Pro IQ (Abbott, Wiesbaden, Germany) on the upper arm for 14 consecutive days, which measured the interstitial glucose concentration in 15-min sampling intervals. Participants were not able to see glucose concentrations, and recorded sensor data were retrieved later by the study assistant with the corresponding reader device. Glycemic control was assessed as following: 24-h mean sensor glucose (MSG) level; maximum and minimum sensor glucose and area under the glucose curve (AUC) calculated using the trapezoidal rule.

The analysis of CGM-based glycemic indices describing intra-day and inter-day GV was performed by the Excel tool EasyGV (27). Next to the mean glucose value, its standard deviation (SD) was assessed to show variation from the glucose average (27). Based on SD, the mean amplitude of glucose excursions (MAGE) was calculated, which describes the height of the glucose excursion when values were higher than 1 SD (27). Moreover, the continuous overlapping net glycemic action (CONGA) was calculated to describe the difference between glucose values at various set intervals. In this study, the CONGA length was set to 60 min. Moreover, the mean of absolute glucose (MAG) change reflecting kinetics of glycemic change per unit of time was calculated using the sum of differences of consecutive glucose levels and was then divided by the total time in hours (28). Next to the mentioned intra-day GV indices, the mean of daily differences (MODD) was calculated to describe inter-day GV based on the average of different glucose values at the same time on different days. Furthermore, the low blood glucose index (LBGI) and high blood glucose index (HBGI) were assessed by Easy GV through the conversion of glucose values to risk scores (LBGI: <0; HBGI: >0). The normal reference range for mean glucose and GV derived from CGM in subjects without diabetes were published previously (27). Furthermore, we manually calculated the coefficient of variation in percentage (CV %) based on formula SD/MSG x 100 as described (24). To achieve an overview of the diurnal glucose profiles, glucose average was calculated at each time point for all days of the study period (intervention or baseline).

HbA1c was assessed using the DCA Vantage Analyzer (Siemens Healthcare, Erlangen, Germany).

### Statistical analysis

For statistical analysis, the software SPSS 25 (IBM, Chicago, IL, United States) was used. The results were expressed as mean ± SD when normally distributed and median (IQR) when not normally distributed. The analysis of the data distribution was performed with the Shapiro–Wilk test. Not normally distributed data were logarithmically transformed before analysis and tested again for the distribution. The within-group comparisons (values after/during the intervention vs. values before the intervention) were assessed by paired Student's *t*-test for normally distributed data or the Wilcoxon test for non-normally distributed data. The between-group comparisons (RS group vs. control group) were conducted for intervention-induced changes (post–pre = Δ values) in each group using Student's unpaired *t*-test for normally distributed data or the Mann–Whitney *U*-test for non-normally distributed data. Comparison of the CGM glucose profiles was performed using the mixed measures ANOVA (anova_test package) by the R software. The statistical significance level was accepted as a *p*-value of <0.05. The visualization of the data was performed using GraphPad Prism software version 5.0 (GraphPad Prism Inc., La Jolla, CA, USA).

## Results

### Baseline characteristics of study population

A total of 21 participants, 8 men and 13 women, with an average age of 53.4 ± 11.5 years, BMI 27.8 ± 5.9 kg/m^2^, and HbA1c 5.60 (5.40–5.85) % were included in the analysis. No participants with known diabetes were enrolled in the trial. The control group included five men and six women aged 55.5 ± 11.6 years, of which two were overweight (BMI > 25 kg/m^2^), five were obese (BMI > 30 kg/m^2^), and four had prediabetes according to HbA1c value (5.7–6.4%). The RS group consisted of three men and seven women aged 51.1 ± 11.6 years, of which four were overweight, 2 were obese, and 4 had prediabetes (Table 1). No significant differences were found between the RS and control groups in the anthropometric measurements (body weight, BMI, total and visceral fat content, waist, and hip circumferences), HbA1c levels, and mean sensor glucose values prior to intervention (Table 1).

**Table 1:** Baseline characteristics of study population.

	**All subjects**	**Reduced-sugar (RS) group**	**Control group**
N	21	10	11
Male	8	3	5
Female	13	7	6
Age (years)	53.4 ± 11.5	51.1 ± 11.6	55.5 ± 11.6
**Ethnicity**			
Caucasian	15	7	8
Turkic	2	1	1
Iranic	1	0	1
Asian	1	0	1
Hispanic	2	2	0
Weight (kg)	83.9 ± 23.1	77.0 ± 14.6	90.1 ± 28.1
BMI (kg/m^2^)	27.8 ± 5.9	26.2 ± 4.0	29.3 ± 7.1
Normal weight/ overweight/obese (*n*)	8/6/7	4/4/2	4/2/5
Fat mass (%)	32.5 ± 10.3	32.6 ± 8.7	32.4 ± 12.0
Visceral fat (%)	8.0 (6.5–12.5)	8.00 (5.75–10.25)	11.00 (7.00–16.00)
Muscle mass (%)	29.7 ± 5.4	29.7 ± 4.6	29.7 ± 6.3
Waist circumference (cm)	94.8 ± 16.7	89.8 ± 12.6	99.4 ± 19.1
Hip circumference (cm)	97.0 (91.5–107.5)	93.5 (92.3–104.5)	98.0 (90–116)
MSG (mmol/L)	5.91 ± 0.49^a^	5.86 ± 0.44^a^	5.96 ± 0.54
HbA1c (%)	5.60 (5.40–5.85)	5.60 (5.38–5.83)	5.60 (5.40–5.10)
Total calories (kcal)	1974 (1782–2098)^b^	1957 (1619–2049)^b^	2015 (1782–2191)
Fat (EN %)	37.9 ± 4.3^b^	37.2 ± 4.9^b^	38.4 ± 3.9
Carbohydrates (EN %)	43.5 ± 4.8^b^	44.8 ± 6.0^b^	42.4 ± 3.5
Sugar (EN %)	14.0 ± 4.6^b^	14.4 ± 5.1^b^	13.8 ± 4.4
Protein (EN %)	15.3 (14.7–18.5)^b^	15.1 (14.7–17.6)^b^	16.1 (14.7–20.8)
Fiber (EN %)	2.00 ± 0.62^b^	2.28 ± 0.59^b^	1.77 ± 0.57

No significant differences were found between the RS and control groups as tested by independent samples t-test for normally distributed data (shown as mean ± SD) or Mann–Whitney U-test for non-normally distributed data [shown as data as median (IQR)]. ^a^One participant was excluded due to unreadable CGM sensor data. ^b^One participant was excluded due to missing nutrition protocols. BMI, body mass index; MSG, mean sensor glucose; AUC_gluc_, area under the glucose curve; SD, standard deviation; CV, coefficient of variation; MAGE, mean amplitude of glucose excursions; CONGA, continuous overall net glycemic action; MAG change, mean absolute glucose change; MODD, mean of daily differences; LBGI, low blood glucose index; HBGI, high blood glucose index; HbA1c, glycated hemoglobin.

### Calorie intake and food composition

**Figure 1:**
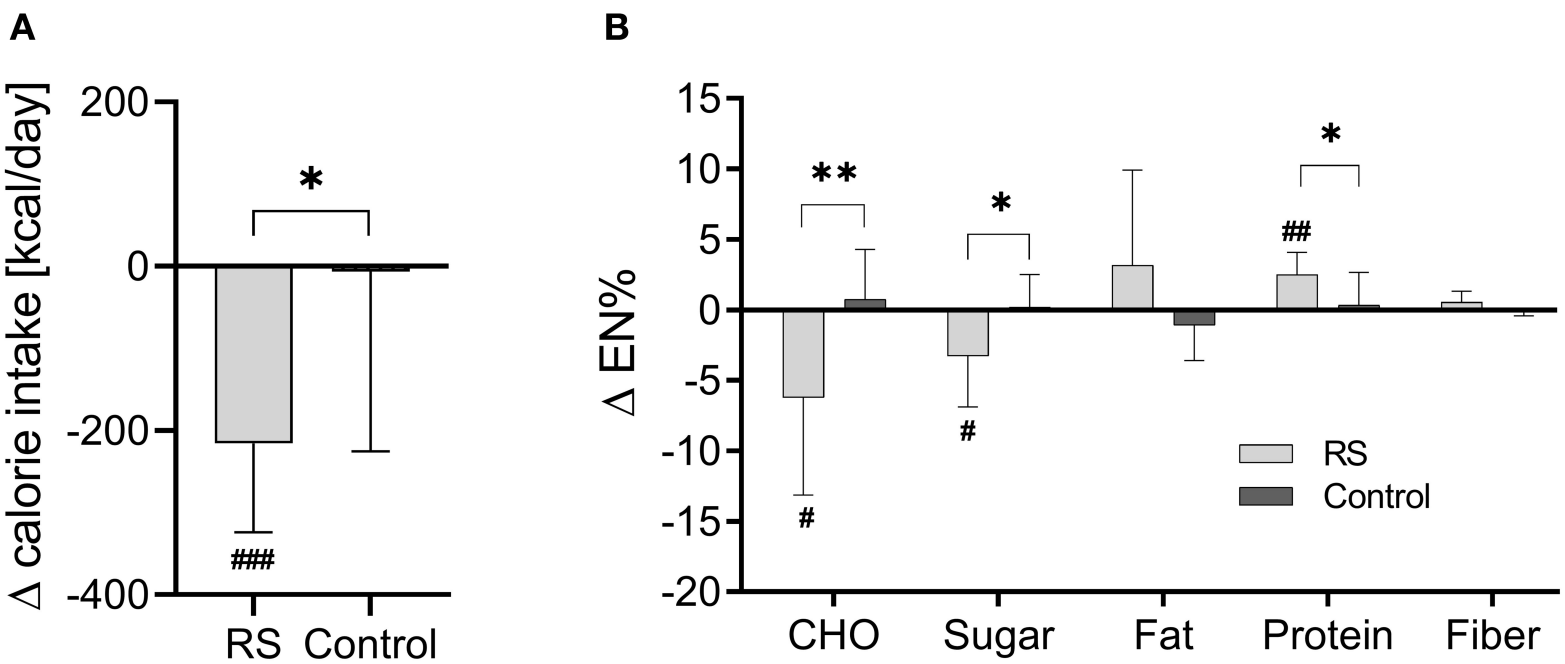
Changes of daily calorie intake **(A)** and macronutrient composition **(B)** during the intervention in the RS and control groups. RS group *n* = 9, control group *n* = 11 (one participant in the RS group was excluded due to missing nutrition protocol). Data are visualized as columns and whiskers for mean ± SD. ^#^*p* < 0.05, ^##^*p* < 0.01, and ^###^*p* < 0.001 in the within-group comparisons of dietary parameters (during the intervention vs. before the intervention) assessed by paired *t*-test. ^*^p < 0.05, ^**^p < 0.01, and ^***^p < 0.001 in the between-group comparisons (RS group vs. control group) of intervention-induced changes (Δ values) assessed by Student's unpaired *t*-test. CHO, carbohydrates.

The calorie intake, macronutrient, and sugar intake did not differ between the RS and control groups prior to intervention (Table 1). They altered during the intervention within the RS group but not within the control group. In the RS group, the calorie intake decreased by 216 ± 108 kcal (*p* = 3.3 × 10^−4^) compared to the baseline phase, and this intervention-induced change (Δ value) was significant in comparison with the control group (*p* = 0.014) (Figure 1A). Furthermore, the RS group substantially reduced the daily sugar intake from 66.6 ± 25.6 g to 44.1 ± 12.9 g (*p* = 0.010) in the within-group comparison to the baseline phase which corresponds to the reduction from 14.4 ± 5.1 EN% to 11.1 ± 3.3 EN% (*p* = 0.026), and this change differed from the control group (*p* = 0.018). The RS group also showed a decrease in total carbohydrate intake (−6.22 ± 6.92 EN%, *p* = 0.027) and an increase of protein intake (+2.51 ± 1.56 EN%, *p* = 0.001) compared to the baseline values, and these changes differed between the groups (*p* = 0.009 and *p* = 0.028, respectively) (Figure 1B). Fat and fiber intake did not change within any group compared to the baseline values and did not differ in the between-group comparison (Figure 1B).

### Anthropometric and body composition parameters

**Figure 2:**
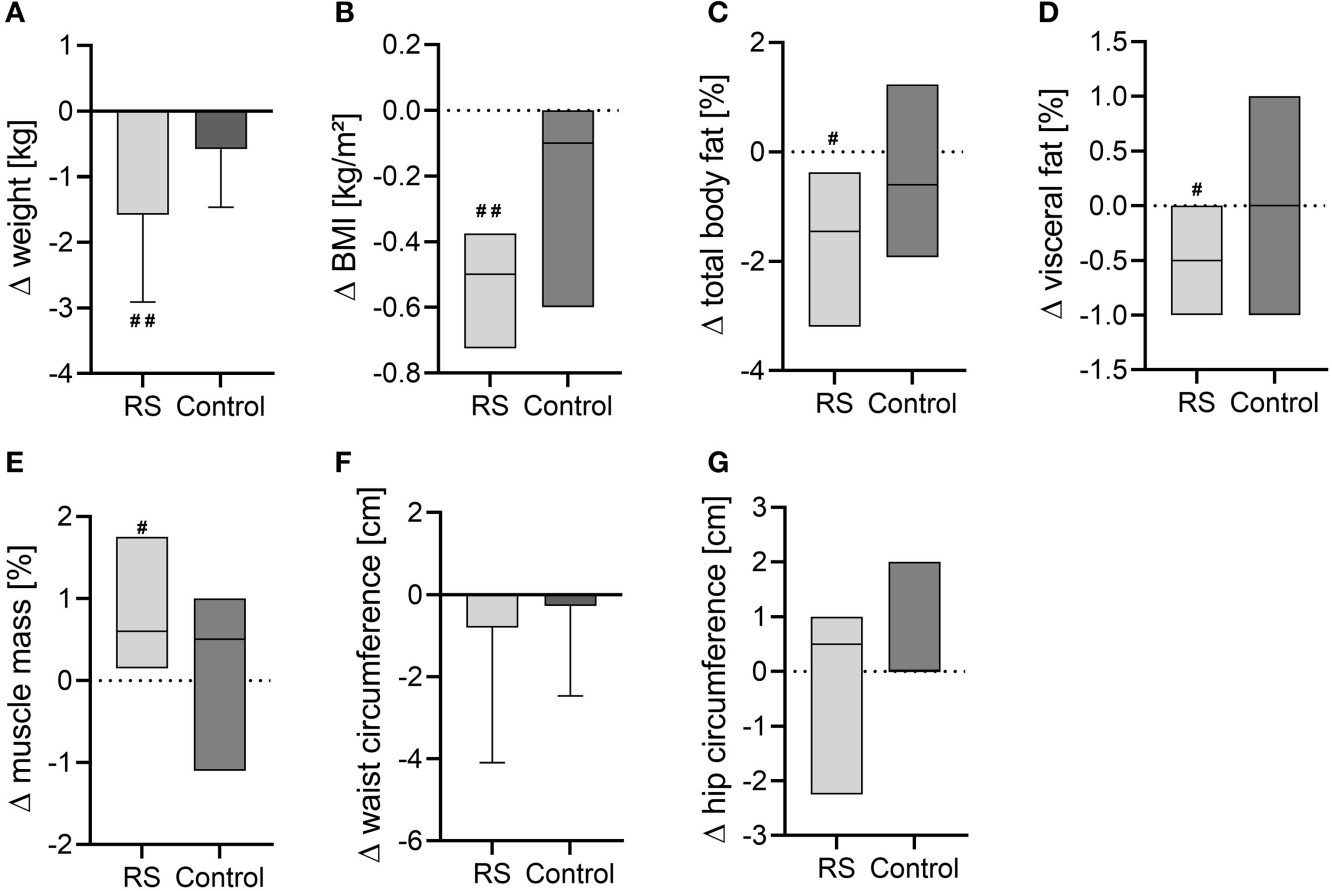
Changes of anthropometric and body composition parameters after the intervention in the RS and control groups. **(A)** Body weight; **(B)** BMI; **(C)** total body fat content; **(D)** visceral fat content; **(E)** muscle mass in percentage; **(F)** waist circumference; **(G)** hip circumference. Normally distributed data are visualized as columns and whiskers for mean ± SD. Non-normally distributed data are shown as box plots for median and IQR (line at median, top of the box at the 75th percentile, bottom of the box at the 25th percentile). ^#^*p* < 0.05, ^*##*^*p* < 0.01 in the within-group comparisons of parameters (after the intervention vs. before the intervention) assessed by paired *t*-test or Wilcoxon test.

In the RS group, weight and BMI decreased after the intervention (weight: −1.58 ± 1.33 kg, *p* = 0.005; BMI: −0.50 (−0.73 to −0.38) kg/m^2^, *p* = 0.005) compared to the baseline values (Figures 2A, B). Along with this, the RS group reduced the total body fat content (*p* = 0.012) and visceral fat content (*p* = 0.015), while the skeletal muscle mass increased (*p* = 0.023) after the intervention compared to the baseline values (Figures 2C–E), but waist and hip circumferences were not affected (Figures 2F, G). In the control group, no anthropometric parameters were altered compared to the baseline phase. However, between-group comparisons of intervention changes were not significant in any of the assessed anthropometric parameters (Figures 2A–G).

### Glycemic parameters

**Figure 3:**
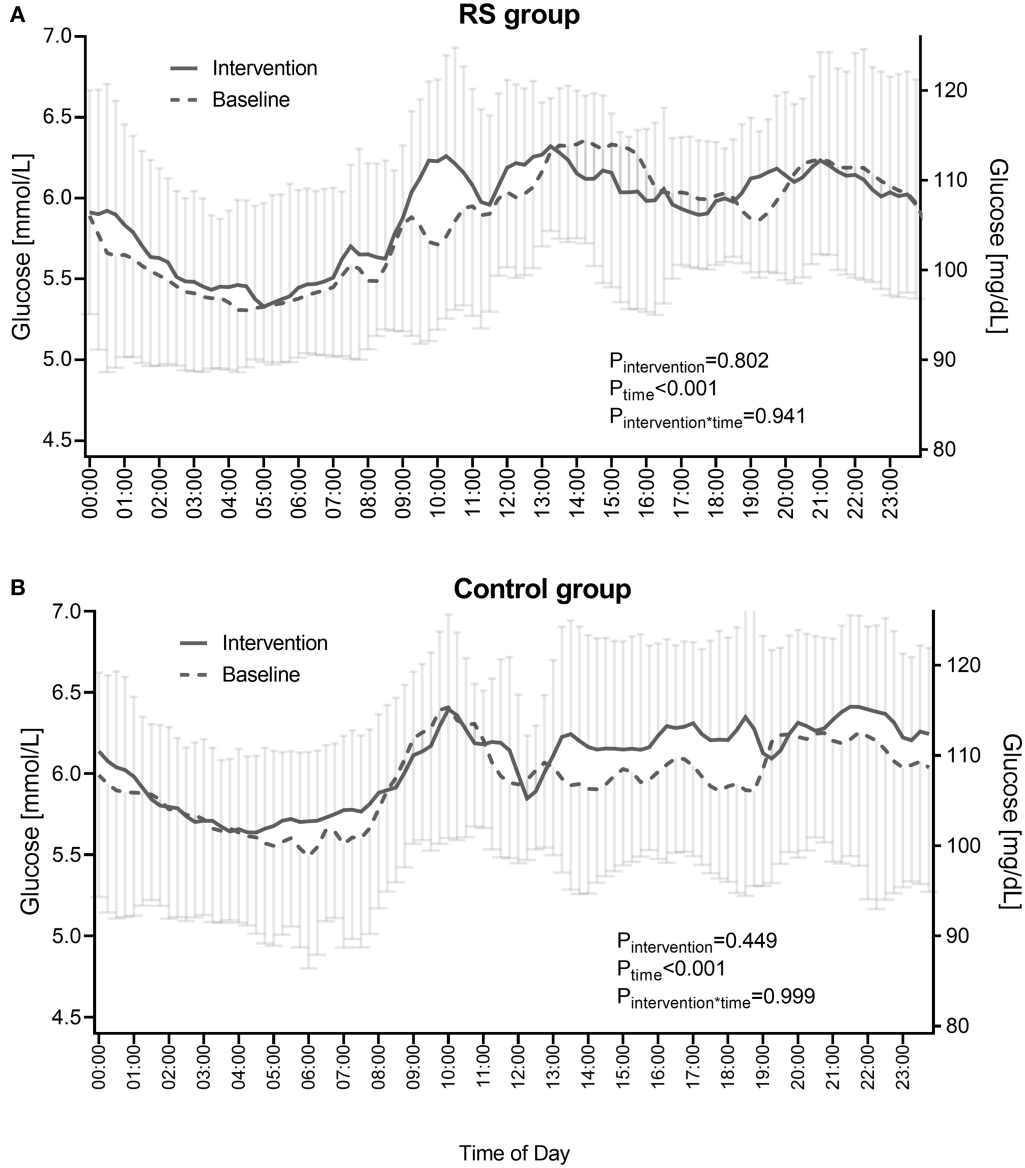
24-h glucose profiles in the RS group **(A)** and control group **(B)** before and during the intervention. RS group *n* = 9, control group *n* = 11 (one participant in the RS group was excluded due to unreadable sensor data). Data points are shown as mean ± SD. *P*-values show the comparison of diurnal glucose profiles between the baseline and intervention phases as calculated by the RM ANOVA.

The 24-h glucose profiles in the RS group also showed no alterations during the intervention compared to the baseline phase as assessed by the mixed measures ANOVA (P_intervention =_ 0.802, P_time_ <0.001, ${\text{P}}_{{\text{intervention}}^{*}}$_time_ = 0.941) (Figure 3A, Supplementary Table 1). Similarly, in the control group, no changes of 24-h glucose profiles were observed (P_intervention =_ 0.449, P_time_ <0.001, ${\text{P}}_{{\text{intervention}}^{*}}$_time_= 0.999) (Figure 3B, Supplementary Table 2).

As assessed by CGM, the mean 24-h glucose and area under the sensor glucose curve (AUC_gluc_) showed no changes during the RS intervention compared to the baseline values (Table 2). Minimum and maximum glucose values did not show changes between the intervention and baseline phases in the RS group (Table 2). In agreement with this, intra-day indices of glycemic variability were not altered in the RS group compared to the baseline values including SD (0.90 ± 0.17 vs. 0.89 ± 0.28 mmol/L), CV (14.8 (13.9–16.8) vs. 13.8 (12.1–15.1) %), MAGE (1.14 ± 0.20 vs. 1.11 ± 0.28 mmol/L), CONGA (5.29 ± 0.34 vs. 5.39 ± 0.37 mmol/L), and MAG change (1.15 ± 0.24 vs. 1.13 ± 0.35 mmol/L/h). The inter-day variability, measured as MODD, also demonstrated no within-group changes during the RS intervention (0.79 ± 0.20 vs. 0.78 ± 0.08 mmol/L). Furthermore, no changes of LBGI (1.17 ± 0.54 vs. 1.14 ± 0.39) and HBGI (0.76 ± 0.40 vs. 0.77 ± 0.37) occurred upon the RS diet vs. baseline phase (Table 2). In agreement with these observations, the HbA1c values remained unchanged after the intervention in the RS group (Table 2). In the control group, as expected, no glycemic parameters were altered compared to the baseline phase. The between-group comparison of intervention-induced changes did not reveal differences in any analyzed glycemic parameter (Table 2).

**Table 2:** Glycemic control and glycemic variability by 24-h continuous glucose monitoring.

	**Reduced-sugar (RS) group**			**Control group**			**Intervention-induced changes (**Δ**)** ^**c**^		
	**Baseline**	**Intervention**	***p*** **-value**	**Baseline**	**Intervention**	***p*** **-value**	**RS**	**Control**	***p*** **-value**
**Glycemic parameters** ^b^									
MSG (mmol/L)	5.86 ± 0.44	5.95 ± 0.37	0.585	5.96 ± 0.54	6.12 ± 0.43	0.364	0.09 ± 0.44	0.16 ± 0.57	0.738
Minimum (mmol/L)	5.12 ± 0.36	5.17 ± 0.63	0.831	5.25 ± 0.56	5.46 ± 0.47	0.338	0.05 ± 0.73	0.21 ± 0.69	0.628
Maximum (mmol/L)	6.84 ± 0.62	6.85 ± 0.38	0.954	6.93 ± 0.84	7.01 ± 0.61	0.729	0.01 ± 0.56	0.08 ± 0.75	0.820
AUC_gluc_ (h x mmol/L)	8383 ± 653	8457 ± 582	0.776	8527 ± 769	8757 ± 815.0	0.372	74.6 ± 760.3	229.7 ± 815.0	0.668
**Glycemic variability** ^a^									
SD (mmol/L)	0.90 ± 0.17	0.89 ± 0.28	0.440	0.85 ± 0.19	0.88 ± 0.12	0.220	−0.02 ± 0.15	0.03 ± 0.10	0.475
CV (%)	14.8 (13.9–16.8)	13.8 (12.1–15.1)	0.173	14.4 ± 3.3	14.3 ± 1.4	0.712	−1.50 (−2.09–0.13)	0.41 (−0.68–1.99)	0.119
MAGE (mmol/L)	1.14 ± 0.20	1.11 ± 0.28	0.505	1.03 ± 0.18	1.09 ± 0.20	0.146	−0.04 ± 0.15	0.06 ± 0.13	0.144
CONGA (mmol/L)	5.29 ± 0.34	5.39 ± 0.37	0.481	5.41 ± 0.48	5.56 ± 0.37	0.376	0.01 ± 0.56	0.08 ± 0.75	0.820
MAG change (mmol/L/h)	1.15 ± 0.24	1.13 ± 0.35	0.768	1.01 (0.96–1.11)	1.05 (0.96–1.20)	0.374	−0.02 ± 0.20	0.03 ± 0.14	0.451
MODD (mmol/L)	0.79 ± 0.20	0.78 ± 0.08	0.561	0.82 ± 0.23	0.79 ± 0.11	0.842	0.01 (−0.08–0.06)	0.01 (−0.06–0.09)	0.970
LBGI	1.17 ± 0.54	1.14 ± 0.39	0.458	1.02 ± 0.68	0.78 ± 0.40	0.289	−0.02 ± 1.25	−0.25 ± 0.73	0.623
HBGI	0.76 ± 0.40	0.77 ± 0.37	0.907	0.77 ± 0.42	0.76 ± 0.29	0.837	0.01 ± 0.25	−0.02 ± 0.27	0.826
HbA1c (%)^b^	5.62 ± 0.25	5.55 ± 0.28	0.271	5.60 (5.40–6.10)	5.60 (5.40–5.80)	0.200	−0.07 ± 0.19	−0.06 ± 0.16	0.934

Within-group comparisons (intervention vs. baseline) were assessed by paired Student's t-test for normally distributed data (shown as mean ± SD) or Wilcoxon test for non-normally distributed data [shown as median (IQR)]. ^a^RS group *n* = 9; control group *n* = 11. ^b^RS group *n* = 10; control group *n* = 11. ^c^Comparison of intervention-induced changes (Δ) between RS and control groups by independent samples t-test for normally distributed data or Mann–Whitney U-test for non-normally distributed data. MSG, mean sensor glucose; AUC_gluc_, area under the glucose curve; SD, standard deviation; CV, coefficient of variation; MAGE, mean amplitude of glucose excursions; CONGA, continuous overall net glycemic action; MAG change, mean absolute glucose change; MODD, mean of daily differences; LBGI, low blood glucose index; HBGI, high blood glucose index; HbA1c, glycated hemoglobin.

## Discussion

The present study is the first trial evaluating the impact of the reduced free sugar consumption on 24-h glucose profiles in non-diabetic individuals. We hypothesized that RS diet would decrease mean 24-h glucose and decline glycemic variation even if subjects do not have diabetes. The main finding of the study was that non-diabetic individuals did not improve mean glucose and glycemic variability despite the reduction of body weight and body fat.

Our initial hypothesis was based on two expectations: (i) Calorie deficit due to the reduced free sugar consumption and corresponding weight loss would improve glycemic control, and (ii) free sugar reduction would decrease PPG peaks and, in this way, improve glycemic variation. The last expectation resulted from the literature on the beneficial effects of reducing total carbohydrates and added sugars intake of subjects with T2D (21–24). Study subjects did not have diabetes, but some of them were obese or overweight and showed prediabetes accordingly to the baseline HbA1c values. However, in non-diabetic individuals, we did not observe any changes of 24-h glucose levels, AUC glucose, minimum and maximum glucose levels, LBGI and HBGI scores, and HbA1c upon the RS diet. We also found no changes of intra-daily glucose variability assessed by SD, CV, MAGE, CONGA, and MAG indices, and no alterations of the intra-day variability measured as MODD index. In agreement with these findings, we also did not observed changes of diurnal glucose pattern upon the RS diet compared with baseline values. Thus, metabolically healthy individuals without manifested changes in glucose regulation (diabetes) might be metabolically flexible enough to provide good glycemic control even upon high sugar consumption. This might explain why no glycemic trait improvements occurred upon sugar reduction in the present study.

Interestingly, we did not observe changes of glycemic traits despite the reduction of body weight and body fat in the RS group. It has to be noted that body weight and body fat may be associated with reduced total energy intake and not with sugar reduction *per se*. Indeed, in the majority of published studies, the link between sugar reduction and improvement of glycemic control can, in a large part, be explained by the RS-induced weight loss. The weight loss due to a calorie deficit (29) has been shown to be an important factor to reduce the risk for T2D (30). Especially sugar is often described as “empty calories” with a deficiency of nutrients (31). Past research referred to an association of added sugar and sugar-sweetened beverages (SSB) with increased visceral, pericardial, and subcutaneous adipose tissue as well as weight gain in children and adults (32–34). Notably, if isoenergetic exchange of sugars and carbohydrates took place, weight changes did not occur (10). Furthermore, the association of sugar intake and T2D risk is eliminated after adjustment for the BMI (32). Most carbohydrate- or sugar-reducing diets improving glycemic control were accompanied by weight loss (21, 23). Intensive weight reduction can even induce diabetes remission (34% of participants with 5–10 kg loss, 57% of participants with 10–15 kg loss, and 86% of participants who lost 15 kg or more) (35). Therefore, promoting weight loss remains a primary nutritional strategy for improving glycemic control in early T2D, although some data show that a short-term carbohydrate reduction can also provide improvements of glycemic control in T2D, independent of weight loss (24). In our study, the sugar reduction in the RS group decreased calorie intake of participant by about 216 kcal and resulted in a minor decrease of BMI (−0.5 kg/m^2^) and body weight (−1.58 kg) within 4 weeks. Therefore, we tend to assume that the positive change of glycemic control will only be achieved by a greater weight reduction.

Interestingly, in the RS group, we observed a shift in macronutrient intake, i.e., the reduction of sugar and carbohydrate intake in favor of protein intake. According to the literature, this shift supports weight loss in terms of hypocaloric diets and the weight maintenance (36). Current evidence describes high-protein diets as more beneficial for weight loss than diets with normal protein intake (37). In particular, in a randomized controlled trial of 132 participants who underwent one of four energy-restricted diets with varying amounts of protein and carbohydrate, the component of high-protein intake was highlighted as the main factor in low-carbohydrate diets (38). Obviously, the change of carbohydrate in favor of protein intake along with calorie reduction contributed to the weight loss in the RS group. Mechanisms explaining beneficial effects of the high-protein diet include changed secretion of incretins (GLP1) and insulin, satiety hormones (e.g., PYY), alterations of lipid metabolism, inflammation, oxidative stress, ER stress, and other hormonal and molecular pathways (39–41). Notably, RS group also showed the change of body composition which can be similarly induced both by energy reduction and by the increased protein intake. Indeed, in study participants, the reduction of general fat content and visceral fat was accompanied by a relative increase in muscle mass percentage. A body of evidence shows that high-protein diets can preserve muscle mass during weight loss although protein quantity and quality remain debatable (42). Notably, the control group did not change dietary composition and energy intake and correspondingly did not change body weight and other anthropometric measures.

Several strengths and limitations of the study have to be mentioned. The main strength is the detailed analysis of the glycemic variation indices upon RS diet which was for the first time conducted using CGM. Another strength of the study is the usage of the Free Style Libre Pro IQ model of glucose sensor which enabled the blinding of the study participants as they did not see their current glucose values and therefore reduced the possibility of unintended bias in food behavior. Finally, the use of food diaries for 14 consequent days during the baseline and intervention phases allowed the high-quality assessment of dietary compliance. A limitation of the study is the relatively moderate reduction of total sugar intake that might result from the increased fruit consumption in the RS group which was recommended as an alternative to sweets but contain intrinsic sugars. Therefore, in future RS trials, a more pronounced sugar intake reduction should be aimed at, through sensitizing individuals to the fact that certain products contain a lot of sugar in their natural state, even if those are no added sugars. Another limitation is the relatively small sample size and heterogeneity of the study population. A more homogenous population of non-diabetic subjects should be recruited for future RS studies, e.g., only obese subjects or only subjects with impaired glucose tolerance.

## Conclusion

In summary, our current investigation showed no direct effect of reducing free sugar intake on mean 24-h glucose and glycemic variability in individuals without diabetes, despite of moderate weight and body fat reduction.

## Data availability statement

The raw data supporting the conclusions of this article will be made available by the authors, without undue reservation.

## Ethics statement

The studies involving humans were approved by Ethics Committee of the Charité-Universitätsmedizin Berlin. The studies were conducted in accordance with the local legislation and institutional requirements. The participants provided their written informed consent to participate in this study.

## Author contributions

CP, HD, JW, and OP-R: conceptualization, writing—reviewing and editing, and resources. CP, BP, JW, and OP-R: methodology. BP: software, formal analysis, visualization, and data curation. CP and BP: investigation. CP and OP-R: project administration. BP and OP-R: writing—original draft preparation. OP-R: supervision and funding acquisition. All authors have read and approved the final manuscript.
