# Supplementary material for: Effects of reducing free sugars on 24-hour glucose profiles and glycemic variability in subjects without diabetes
Source: Front Nutr. 2023 Oct 2;10:1213661. doi: 10.3389/fnut.2023.1213661 (PMC10577299; doi:10.3389/fnut.2023.1213661)
Supplement: Supplementary file 1 [file Data_Sheet_1.pdf]

SUPPLEMENTAL TABLE 1: GLUCOSE DATA DISTRIBUTION IN THE RS GROUP AS MEASURED BY 24-HOURS CONTINOUS GLUCOSE MONITORING

experiment phase	timepoint	variable	statistic	p
intervention	00_00	Glucose	0.92200891	0.40913137
intervention	00_15	Glucose	0.98097076	0.96908145
intervention	00_30	Glucose	0.96617341	0.86016897
intervention	00_45	Glucose	0.94923283	0.68163988
intervention	01_00	Glucose	0.95257948	0.71837964
intervention	01_15	Glucose	0.9582703	0.78005214
intervention	01_30	Glucose	0.93472496	0.52759219
intervention	01_45	Glucose	0.95687756	0.76514105
intervention	02_00	Glucose	0.95925225	0.79046081
intervention	02_15	Glucose	0.92396255	0.42605256
intervention	02_30	Glucose	0.90824798	0.30380403
intervention	02_45	Glucose	0.91091461	0.32232849
intervention	03_00	Glucose	0.90431561	0.27808966
intervention	03_15	Glucose	0.9277294	0.46003003
intervention	03_30	Glucose	0.97242844	0.91466292
intervention	03_45	Glucose	0.96574034	0.85604769
intervention	04_00	Glucose	0.9155247	0.35647759
intervention	04_15	Glucose	0.83699798	0.05347454
intervention	04_30	Glucose	0.85596181	0.08663707
intervention	04_45	Glucose	0.86434324	0.10686834
intervention	05_00	Glucose	0.86362518	0.10497403
intervention	05_15	Glucose	0.85553465	0.08571003
intervention	05_30	Glucose	0.85497297	0.08450538
intervention	05_45	Glucose	0.83603246	0.05216434
intervention	06_00	Glucose	0.86981938	0.12240688
intervention	06_15	Glucose	0.85281669	0.08002881
intervention	06_30	Glucose	0.84677141	0.06865369
intervention	06_45	Glucose	0.81000465	0.02654869
intervention	07_00	Glucose	0.83977944	0.05742906
intervention	07_15	Glucose	0.95105346	0.70163349
intervention	07_30	Glucose	0.9635556	0.83470378
intervention	07_45	Glucose	0.97523189	0.93540176
intervention	08_00	Glucose	0.90975274	0.31414776
intervention	08_15	Glucose	0.89187427	0.20855747
intervention	08_30	Glucose	0.88490584	0.17672491
intervention	08_45	Glucose	0.98749569	0.9916633
intervention	09_00	Glucose	0.96865588	0.88299562
intervention	09_15	Glucose	0.98891247	0.99442651
intervention	09_30	Glucose	0.98186882	0.97319435
intervention	09_45	Glucose	0.88859087	0.19296988
intervention	10_00	Glucose	0.95148835	0.70640882
intervention	10_15	Glucose	0.9507607	0.69841816
intervention	10_30	Glucose	0.88090088	0.16048121
intervention	10_45	Glucose	0.87426357	0.13654157
intervention	11_00	Glucose	0.89546998	0.22689778
intervention	11_15	Glucose	0.88544319	0.17901424

intervention	11_30	Glucose	0.79848385	0.01962274
intervention	11_45	Glucose	0.85696259	0.0888465
intervention	12_00	Glucose	0.94198738	0.60296824
intervention	12_15	Glucose	0.9370768	0.55148663
intervention	12_30	Glucose	0.92780942	0.4607707
intervention	12_45	Glucose	0.94142906	0.59702003
intervention	13_00	Glucose	0.96179028	0.81686294
intervention	13_15	Glucose	0.95133861	0.70476479
intervention	13_30	Glucose	0.94390108	0.62350977
intervention	13_45	Glucose	0.91542125	0.35568148
intervention	14_00	Glucose	0.93112528	0.492134
intervention	14_15	Glucose	0.95340674	0.72743903
intervention	14_30	Glucose	0.97490912	0.93314762
intervention	14_45	Glucose	0.95530218	0.74810596
intervention	15_00	Glucose	0.94661139	0.65293546
intervention	15_15	Glucose	0.95463153	0.74081116
intervention	15_30	Glucose	0.97539654	0.93653778
intervention	15_45	Glucose	0.94583833	0.64450953
intervention	16_00	Glucose	0.94957278	0.68537175
intervention	16_15	Glucose	0.87599643	0.14245197
intervention	16_30	Glucose	0.91271494	0.33534172
intervention	16_45	Glucose	0.76678343	0.00847994
intervention	17_00	Glucose	0.82201595	0.03631183
intervention	17_15	Glucose	0.85542762	0.08547923
intervention	17_30	Glucose	0.8807362	0.15984337
intervention	17_45	Glucose	0.89177821	0.20808598
intervention	18_00	Glucose	0.91950038	0.38811925
intervention	18_15	Glucose	0.89438894	0.22124028
intervention	18_30	Glucose	0.94114099	0.59395963
intervention	18_45	Glucose	0.97487017	0.93287317
intervention	19_00	Glucose	0.94427978	0.62760025
intervention	19_15	Glucose	0.87326242	0.13323115
intervention	19_30	Glucose	0.84382316	0.06368486
intervention	19_45	Glucose	0.90096848	0.2576642
intervention	20_00	Glucose	0.95917593	0.78965532
intervention	20_15	Glucose	0.9605164	0.80371013
intervention	20_30	Glucose	0.98235477	0.97528498
intervention	20_45	Glucose	0.97040163	0.89814235
intervention	21_00	Glucose	0.93632	0.54373871
intervention	21_15	Glucose	0.96421294	0.84121757
intervention	21_30	Glucose	0.94560687	0.64199137
intervention	21_45	Glucose	0.93417851	0.52211987
intervention	22_00	Glucose	0.9704573	0.89861192
intervention	22_15	Glucose	0.97585604	0.93965756
intervention	22_30	Glucose	0.97802989	0.95338043
intervention	22_45	Glucose	0.9366551	0.54716264
intervention	23_00	Glucose	0.91165326	0.32761794
intervention	23_15	Glucose	0.91213288	0.33108942
intervention	23_30	Glucose	0.91223062	0.33180047

intervention	23_45	Glucose	0.89715553	0.23597123
baseline	00_00	Glucose	0.97935419	0.96086895
baseline	00_15	Glucose	0.94164049	0.59927002
baseline	00_30	Glucose	0.92454608	0.43120041
baseline	00_45	Glucose	0.9316242	0.4969635
baseline	01_00	Glucose	0.91782595	0.37454432
baseline	01_15	Glucose	0.89946982	0.24894149
baseline	01_30	Glucose	0.90335167	0.27207173
baseline	01_45	Glucose	0.93828736	0.56399065
baseline	02_00	Glucose	0.94716013	0.65892933
baseline	02_15	Glucose	0.91442987	0.34812165
baseline	02_30	Glucose	0.90920287	0.3103352
baseline	02_45	Glucose	0.93151762	0.49592944
baseline	03_00	Glucose	0.97243767	0.91473535
baseline	03_15	Glucose	0.96676617	0.86574601
baseline	03_30	Glucose	0.87466711	0.13789733
baseline	03_45	Glucose	0.8452796	0.06609468
baseline	04_00	Glucose	0.89550053	0.22705947
baseline	04_15	Glucose	0.9429555	0.6133318
baseline	04_30	Glucose	0.89997333	0.25184341
baseline	04_45	Glucose	0.90746807	0.29855359
baseline	05_00	Glucose	0.89450713	0.22185269
baseline	05_15	Glucose	0.87209077	0.12945156
baseline	05_30	Glucose	0.94156005	0.59841363
baseline	05_45	Glucose	0.95371931	0.73085687
baseline	06_00	Glucose	0.90672629	0.2936294
baseline	06_15	Glucose	0.93497676	0.53012412
baseline	06_30	Glucose	0.92414773	0.42768155
baseline	06_45	Glucose	0.90820632	0.30352169
baseline	07_00	Glucose	0.89673656	0.23368691
baseline	07_15	Glucose	0.92510557	0.43617636
baseline	07_30	Glucose	0.89598601	0.22964273
baseline	07_45	Glucose	0.90180236	0.26262977
baseline	08_00	Glucose	0.92388885	0.42540547
baseline	08_15	Glucose	0.79983218	0.02033113
baseline	08_30	Glucose	0.9467624	0.65458395
baseline	08_45	Glucose	0.99054356	0.99678267
baseline	09_00	Glucose	0.96082327	0.80689793
baseline	09_15	Glucose	0.92626733	0.44663348
baseline	09_30	Glucose	0.91092432	0.32239762
baseline	09_45	Glucose	0.97204646	0.91164245
baseline	10_00	Glucose	0.90049982	0.25490874
baseline	10_15	Glucose	0.92173806	0.40682382
baseline	10_30	Glucose	0.92892608	0.47118778
baseline	10_45	Glucose	0.89667809	0.2333696
baseline	11_00	Glucose	0.91929936	0.38647044
baseline	11_15	Glucose	0.95322987	0.72550363
baseline	11_30	Glucose	0.95747086	0.77151243
baseline	11_45	Glucose	0.9333788	0.51416783

baseline	12_00	Glucose	0.93428087	0.5231426
baseline	12_15	Glucose	0.92752853	0.45817403
baseline	12_30	Glucose	0.94262905	0.60983047
baseline	12_45	Glucose	0.89250043	0.21165397
baseline	13_00	Glucose	0.86454787	0.10741405
baseline	13_15	Glucose	0.83203653	0.04706456
baseline	13_30	Glucose	0.81167738	0.02773579
baseline	13_45	Glucose	0.90673291	0.29367301
baseline	14_00	Glucose	0.95023379	0.69263082
baseline	14_15	Glucose	0.93693265	0.55000672
baseline	14_30	Glucose	0.9273839	0.45684076
baseline	14_45	Glucose	0.92298556	0.41752991
baseline	15_00	Glucose	0.92785072	0.46115335
baseline	15_15	Glucose	0.91823492	0.37782657
baseline	15_30	Glucose	0.88703838	0.18596898
baseline	15_45	Glucose	0.84253007	0.0616163
baseline	16_00	Glucose	0.82415776	0.03838776
baseline	16_15	Glucose	0.87038749	0.12413456
baseline	16_30	Glucose	0.91678812	0.36631197
baseline	16_45	Glucose	0.9527593	0.72035037
baseline	17_00	Glucose	0.94559233	0.64183317
baseline	17_15	Glucose	0.87422188	0.13640219
baseline	17_30	Glucose	0.88697011	0.18566639
baseline	17_45	Glucose	0.86054657	0.09720534
baseline	18_00	Glucose	0.86131084	0.09908162
baseline	18_15	Glucose	0.89801731	0.24073102
baseline	18_30	Glucose	0.88335784	0.17027716
baseline	18_45	Glucose	0.8581239	0.09147737
baseline	19_00	Glucose	0.81319857	0.02886036
baseline	19_15	Glucose	0.87436841	0.13689261
baseline	19_30	Glucose	0.95274487	0.72019219
baseline	19_45	Glucose	0.95553017	0.75058056
baseline	20_00	Glucose	0.95672487	0.76349713
baseline	20_15	Glucose	0.96233183	0.82238676
baseline	20_30	Glucose	0.88989989	0.19905526
baseline	20_45	Glucose	0.92810506	0.46351409
baseline	21_00	Glucose	0.96679387	0.86600474
baseline	21_15	Glucose	0.93134491	0.49425651
baseline	21_30	Glucose	0.86219941	0.10130606
baseline	21_45	Glucose	0.8887688	0.19378712
baseline	22_00	Glucose	0.8787064	0.15216985
baseline	22_15	Glucose	0.90475524	0.28087121
baseline	22_30	Glucose	0.92417937	0.42796033
baseline	22_45	Glucose	0.93609894	0.54148584
baseline	23_00	Glucose	0.95239346	0.7163402
baseline	23_15	Glucose	0.9454737	0.64054354
baseline	23_30	Glucose	0.97637476	0.94308922
baseline	23_45	Glucose	0.96973148	0.89242319

SUPPLEMENTAL TABLE 2: GLUCOSE DATA DISTRIBUTION IN THE CONTROL GROUP AS MEASURED BY 24-HOURS CONTINOUS GLUCOSE MONITORING

experiment phase	timepoint	variable	statistic	p
intervention	00_00	Glucose	0.93200715	0.43151334
intervention	00_15	Glucose	0.94183335	0.54228578
intervention	00_30	Glucose	0.93619147	0.47681087
intervention	00_45	Glucose	0.9244542	0.3575001
intervention	01_00	Glucose	0.93755267	0.49216845
intervention	01_15	Glucose	0.90082431	0.18918172
intervention	01_30	Glucose	0.92167616	0.33284876
intervention	01_45	Glucose	0.98291076	0.98016643
intervention	02_00	Glucose	0.9661858	0.84564474
intervention	02_15	Glucose	0.95956475	0.76624769
intervention	02_30	Glucose	0.95642504	0.72644623
intervention	02_45	Glucose	0.95261778	0.67753659
intervention	03_00	Glucose	0.95347365	0.68854749
intervention	03_15	Glucose	0.9715149	0.90139681
intervention	03_30	Glucose	0.97974734	0.9647416
intervention	03_45	Glucose	0.97176236	0.90374218
intervention	04_00	Glucose	0.95388497	0.69383954
intervention	04_15	Glucose	0.93694658	0.48529391
intervention	04_30	Glucose	0.94109894	0.5335028
intervention	04_45	Glucose	0.90103596	0.19030834
intervention	05_00	Glucose	0.90740962	0.22718563
intervention	05_15	Glucose	0.936853	0.48423765
intervention	05_30	Glucose	0.89023369	0.14000632
intervention	05_45	Glucose	0.89926548	0.18106818
intervention	06_00	Glucose	0.88869836	0.13395221
intervention	06_15	Glucose	0.89506088	0.1607402
intervention	06_30	Glucose	0.91271625	0.26255027
intervention	06_45	Glucose	0.9374678	0.49120234
intervention	07_00	Glucose	0.91981683	0.31711746
intervention	07_15	Glucose	0.90013476	0.1855529
intervention	07_30	Glucose	0.88582702	0.1232828
intervention	07_45	Glucose	0.86957244	0.07654664
intervention	08_00	Glucose	0.86941619	0.0761933
intervention	08_15	Glucose	0.88204835	0.11045866
intervention	08_30	Glucose	0.87361939	0.0862691
intervention	08_45	Glucose	0.88781014	0.13056252
intervention	09_00	Glucose	0.94203651	0.54472803
intervention	09_15	Glucose	0.95063582	0.65208842
intervention	09_30	Glucose	0.90507358	0.2129898
intervention	09_45	Glucose	0.94224756	0.54727086
intervention	10_00	Glucose	0.97196673	0.90566083
intervention	10_15	Glucose	0.95552901	0.71496724
intervention	10_30	Glucose	0.93665184	0.48197194
intervention	10_45	Glucose	0.94173661	0.54112478
intervention	11_00	Glucose	0.94870611	0.62746439

intervention	11_15	Glucose	0.87401101	0.08727062
intervention	11_30	Glucose	0.94733488	0.61011036
intervention	11_45	Glucose	0.96367331	0.81651712
intervention	12_00	Glucose	0.96366909	0.81646694
intervention	12_15	Glucose	0.90288213	0.20039544
intervention	12_30	Glucose	0.87188168	0.08195689
intervention	12_45	Glucose	0.89396754	0.1558093
intervention	13_00	Glucose	0.88755137	0.12959022
intervention	13_15	Glucose	0.91144872	0.25369693
intervention	13_30	Glucose	0.91599246	0.28666134
intervention	13_45	Glucose	0.92091448	0.32633034
intervention	14_00	Glucose	0.93262492	0.43801381
intervention	14_15	Glucose	0.94187419	0.54277628
intervention	14_30	Glucose	0.9366271	0.48169372
intervention	14_45	Glucose	0.94247407	0.55000624
intervention	15_00	Glucose	0.97458341	0.92868036
intervention	15_15	Glucose	0.97924288	0.9618058
intervention	15_30	Glucose	0.949845	0.64197326
intervention	15_45	Glucose	0.96749484	0.86017961
intervention	16_00	Glucose	0.91485658	0.27809867
intervention	16_15	Glucose	0.94021612	0.52304226
intervention	16_30	Glucose	0.90650648	0.22160113
intervention	16_45	Glucose	0.92270708	0.34183569
intervention	17_00	Glucose	0.94076646	0.52955058
intervention	17_15	Glucose	0.93869937	0.5053305
intervention	17_30	Glucose	0.9510231	0.65705186
intervention	17_45	Glucose	0.9669905	0.85463709
intervention	18_00	Glucose	0.96273064	0.80523295
intervention	18_15	Glucose	0.92045842	0.32247664
intervention	18_30	Glucose	0.8949368	0.16017345
intervention	18_45	Glucose	0.91543663	0.28244405
intervention	19_00	Glucose	0.96413594	0.82198927
intervention	19_15	Glucose	0.97284744	0.91373469
intervention	19_30	Glucose	0.98634139	0.99124806
intervention	19_45	Glucose	0.98286476	0.97997881
intervention	20_00	Glucose	0.99109132	0.99844561
intervention	20_15	Glucose	0.97597151	0.93965069
intervention	20_30	Glucose	0.97661867	0.94445339
intervention	20_45	Glucose	0.97440689	0.92722148
intervention	21_00	Glucose	0.93199094	0.43134374
intervention	21_15	Glucose	0.9142943	0.27394031
intervention	21_30	Glucose	0.95310165	0.68376111
intervention	21_45	Glucose	0.93884802	0.50705137
intervention	22_00	Glucose	0.925595	0.36802406
intervention	22_15	Glucose	0.91792711	0.30175177
intervention	22_30	Glucose	0.90667396	0.22262747
intervention	22_45	Glucose	0.93008425	0.41170648
intervention	23_00	Glucose	0.90143812	0.19246586
intervention	23_15	Glucose	0.91992766	0.31803797

intervention	23_30	Glucose	0.9356558	0.47084877
intervention	23_45	Glucose	0.8988516	0.17896773
baseline	00_00	Glucose	0.87428385	0.08797497
baseline	00_15	Glucose	0.90378715	0.20551355
baseline	00_30	Glucose	0.89145256	0.14499335
baseline	00_45	Glucose	0.86437242	0.06559811
baseline	01_00	Glucose	0.88524056	0.12120425
baseline	01_15	Glucose	0.89733266	0.1714481
baseline	01_30	Glucose	0.87096402	0.0797642
baseline	01_45	Glucose	0.85285083	0.04649062
baseline	02_00	Glucose	0.86608676	0.06902826
baseline	02_15	Glucose	0.90326849	0.20256625
baseline	02_30	Glucose	0.90564924	0.21641299
baseline	02_45	Glucose	0.86598334	0.06881648
baseline	03_00	Glucose	0.87780026	0.09755475
baseline	03_15	Glucose	0.91168096	0.25529956
baseline	03_30	Glucose	0.93859483	0.50412226
baseline	03_45	Glucose	0.96135707	0.7885033
baseline	04_00	Glucose	0.95514257	0.71000696
baseline	04_15	Glucose	0.88374765	0.11606133
baseline	04_30	Glucose	0.89602468	0.16520568
baseline	04_45	Glucose	0.92428741	0.35598108
baseline	05_00	Glucose	0.90989958	0.24322695
baseline	05_15	Glucose	0.91691959	0.29381286
baseline	05_30	Glucose	0.90583231	0.21751186
baseline	05_45	Glucose	0.92458542	0.35869869
baseline	06_00	Glucose	0.93294343	0.44139114
baseline	06_15	Glucose	0.91629773	0.28899989
baseline	06_30	Glucose	0.87370046	0.08647553
baseline	06_45	Glucose	0.86754767	0.07208803
baseline	07_00	Glucose	0.9356611	0.47090746
baseline	07_15	Glucose	0.9303813	0.41472372
baseline	07_30	Glucose	0.93742308	0.49069371
baseline	07_45	Glucose	0.93725531	0.48878844
baseline	08_00	Glucose	0.89337855	0.15321132
baseline	08_15	Glucose	0.90430972	0.20852183
baseline	08_30	Glucose	0.91748249	0.29822675
baseline	08_45	Glucose	0.88464048	0.11911171
baseline	09_00	Glucose	0.90970349	0.24192872
baseline	09_15	Glucose	0.97107874	0.89720462
baseline	09_30	Glucose	0.97074368	0.8939347
baseline	09_45	Glucose	0.93857698	0.5039162
baseline	10_00	Glucose	0.94578153	0.59063822
baseline	10_15	Glucose	0.97053415	0.89186847
baseline	10_30	Glucose	0.93742593	0.49072615
baseline	10_45	Glucose	0.83507392	0.02721638
baseline	11_00	Glucose	0.8974044	0.17179669
baseline	11_15	Glucose	0.92434534	0.35650807
baseline	11_30	Glucose	0.90588002	0.21779901

baseline	11_45	Glucose	0.86436358	0.06558086
baseline	12_00	Glucose	0.88799475	0.13126037
baseline	12_15	Glucose	0.91928303	0.31271378
baseline	12_30	Glucose	0.93197507	0.43117762
baseline	12_45	Glucose	0.91313173	0.26550929
baseline	13_00	Glucose	0.9087283	0.23556179
baseline	13_15	Glucose	0.86335314	0.06363777
baseline	13_30	Glucose	0.7601957	0.00282457
baseline	13_45	Glucose	0.78878399	0.00669759
baseline	14_00	Glucose	0.8218535	0.0182431
baseline	14_15	Glucose	0.83420527	0.02651124
baseline	14_30	Glucose	0.89893093	0.17936861
baseline	14_45	Glucose	0.90448385	0.20953293
baseline	15_00	Glucose	0.92780113	0.38903958
baseline	15_15	Glucose	0.89677715	0.16877094
baseline	15_30	Glucose	0.88138144	0.10833071
baseline	15_45	Glucose	0.95109896	0.65802474
baseline	16_00	Glucose	0.92731425	0.38432642
baseline	16_15	Glucose	0.88555738	0.12232299
baseline	16_30	Glucose	0.84085259	0.03240385
baseline	16_45	Glucose	0.86309294	0.06314652
baseline	17_00	Glucose	0.9065125	0.22163791
baseline	17_15	Glucose	0.93651313	0.48041325
baseline	17_30	Glucose	0.93972537	0.51727498
baseline	17_45	Glucose	0.91070064	0.2485933
baseline	18_00	Glucose	0.87336014	0.0856122
baseline	18_15	Glucose	0.80574619	0.01119682
baseline	18_30	Glucose	0.85112633	0.04414577
baseline	18_45	Glucose	0.89842856	0.17684378
baseline	19_00	Glucose	0.79976857	0.00934145
baseline	19_15	Glucose	0.76912329	0.00369683
baseline	19_30	Glucose	0.7506016	0.00211649
baseline	19_45	Glucose	0.7966806	0.00850705
baseline	20_00	Glucose	0.85125482	0.04431639
baseline	20_15	Glucose	0.81301055	0.01395482
baseline	20_30	Glucose	0.87802142	0.09818951
baseline	20_45	Glucose	0.93658683	0.48124097
baseline	21_00	Glucose	0.92507954	0.36323991
baseline	21_15	Glucose	0.85476021	0.04922951
baseline	21_30	Glucose	0.87591655	0.09230518
baseline	21_45	Glucose	0.85242018	0.04589385
baseline	22_00	Glucose	0.89455618	0.15844643
baseline	22_15	Glucose	0.89383536	0.15522275
baseline	22_30	Glucose	0.88639902	0.12534235
baseline	22_45	Glucose	0.91807232	0.30291038
baseline	23_00	Glucose	0.94250492	0.55037922
baseline	23_15	Glucose	0.86872789	0.07465542
baseline	23_30	Glucose	0.82631774	0.02088381
baseline	23_45	Glucose	0.83782088	0.02957084
